# Demographic dynamics between 5500 and 3500 calBP (3550–1550 BCE) in selected study regions of Central Europe and the role of regional climate influences

**DOI:** 10.1371/journal.pone.0291956

**Published:** 2023-10-25

**Authors:** Ralph Großmann, Mara Weinelt, Johannes Müller

**Affiliations:** 1 Institute of Pre- and Protohistoric Archaeology, Kiel University, Kiel, Germany; 2 Cluster of Excellence ROOTS—Social, Environmental, and Cultural Connectivity in Past Societies, Kiel University, Kiel, Germany; University of California Santa Cruz, UNITED STATES

## Abstract

With their rich Late Neolithic to Early Bronze Age archives, the Circumharz region, the Czech Republic/Lower Austria region, and the Northern Alpine Foreland are well-suited for research on potential links between human activities and climate fluctuations of this period with pronounced archaeological changes. In this paper, we reconstruct the rate and density of the available ^14^C data from 5500 to 3500 calBP (3550–1550 BCE). We ask to what extent population patterns varied over time and space, and whether fluctuations in human populations and their activities varied with local/regional climate changes. To answer these questions, we have compiled an extensive list of published radiocarbon dates and created ^14^C sum calibrations for each region. We also compare population dynamics with local and regional palaeoclimate records derived from high-resolution speleothems. At the regional scale, the results suggest a causal relationship between regional climate and population trends. Climate and associated environmental changes were thus at least partly responsible for demographic trends. These results also allow us to question the motivation for the construction of so-called “Early Bronze Age princely tombs” in the Circumharz region during a period of population decline. Among other things, it can be argued that the upper echelons of society may have benefited from trade relations. However, this process was accompanied by ecological stress, a cooling of the winter climate, a decline in the total population and an increase in social inequality.

## Introduction

In recent years, there has been increasing interest in studying past demographic patterns to understand how population growth and decline are associated with cultural change [1–5], increases or decreases in social complexity [6, 7], and/or changes in food production [3, 8, 9]. In this context, summed probability distributions (SPD) of calibrated archaeological radiocarbon dates are of particular importance and have been applied to a wide range of regional (Iberian Peninsula: [10, 11]; Italy and Levant: [12, 13]; Czech Republic: [14]; Ireland: [15]; Norway: [16]; Southern Germany/Switzerland: [17]; Japan: [18]; Mediterranean: [19]; Central Balkans: [20, 21] and transregional or pan-European studies: [3, 22–27].

The ability to base such studies on absolute calendar chronologies rather than the conventional relative chronologies of archaeological sources also offers the advantage to assess the relationship between population dynamics of different regions and climatic changes on synchronous time scales. Climatic changes often invoked to have caused social and / or economic changes [8, 11, 13, 19, 28–32].

However, criticism of this proxy, which is indeed quite vulnerable to various “sources of interference”, such as the shape of past actions/action changes and the influence of research strategies and questions on sum calibrations (cf. [33], has also led to a number of methodological studies that demonstrate its limitations but also more precisely define its statistical accuracy and applicability (cf. also the response to Torfing by Timpson et al. [34].

In this paper, we will discuss SPD-calibrated radiocarbon dates from several regions and correlate them with climate curves derived from speleothems to draw inferences about human-environment relationships. Recent studies based on broadened interdisciplinary approaches (e.g. [11, 13, 35] constitute an important endeavour by examining archaeological and historical “laboratories” under the basic assumption that humans and the environment have profoundly shaped each other. Studying the effects of environmental change on past populations is an important endeavor to trace the roots of human-environment connectivity in light of contemporary experiences with climate change. These are targeted at exploring questions of social inequality and of environmental hazards (drought, extreme wetness, extreme cold) for past societies, in particular. In general, we understand social inequality as unequal access to resources. However, its form may vary from one society to another and may be visible in different ways in the archaeological and bio-anthropological record [36, 37]. In the European Early Bronze Age, for example, archaeologists have shown that inequality is visible through differential access to burial structures and grave goods, and is also associated with unequal access to certain diets during lifetime [36, 38, 39].

**Fig 1:**
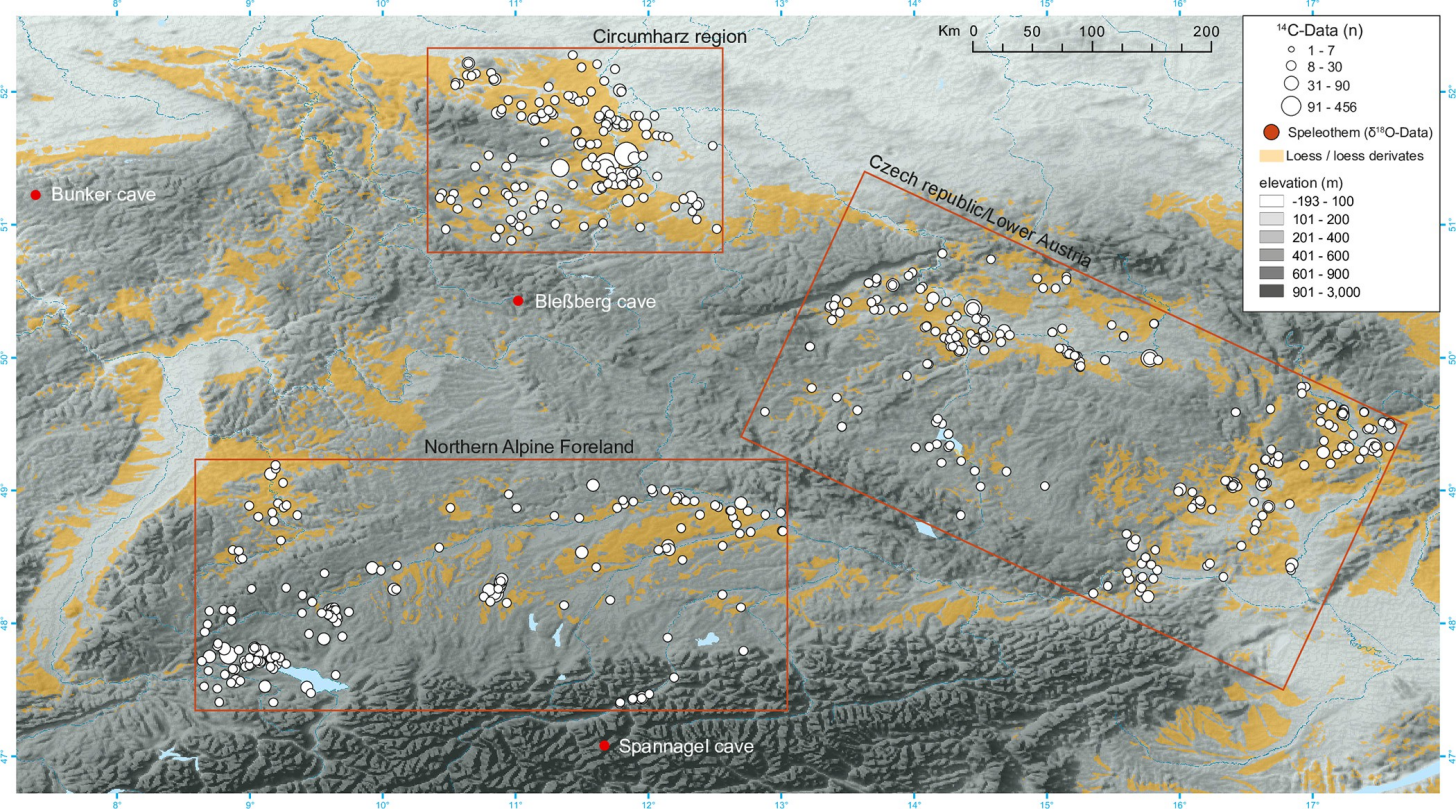
Map showing the distribution of sites with radiocarbon dates and palaeoclimate records. Map based on a digital elevation model for the world (GTOPO30), developed by United States Geological Survey (USGS) [52]. Added are also loess occurrences according to Haase et al. and Lehmkuhl et al. [50, 53]. Geodata of European loess domains is published under the Creative Commons Attribution License (CC BY 4.0). Software: ArcMap 10.8.1.

For our case studies, we chose three regions in Central Europe with rich archaeological sources, ^14^C data, and high-resolution time series of climate variations (Fig 1). The prehistoric Circumharz region, the Czech Republic/Lower Austria region, and the Northern Alpine Foreland are excellent for case studies to investigate regional demographic trends and their response to climate variability under different environmental conditions. For this study, we deliberately selected only high-resolution palaeoclimatic datasets from speleothems located in close spatial proximity to the archaeological study areas, so that direct relevance can be assumed.

In order to answer the question to what extent Central European population patterns fluctuated during the Neolithic-Bronze Age transition and what role climatic changes played in this process, we compiled an extensive dataset of radiocarbon dates (n = 3428) published in various databases and individual publications (S1 Data) and used it to infer demographic trends.

The focus is on the period from the Late Neolithic to the Middle Bronze Age (5500–3500 calBP; 3550–1550 BCE). This spans a series of known archaeological transformations (e.g., the emergence of the Beaker Phenomenon, Early Bronze Age) that occurred in the context of a major climatic reorganization at the end of the HTM (Holocene Thermal Maximum, cf. [40]. We deliberately choose a manageable study period in order to achieve a high and detailed resolution of human-environment relationships and to better link population dynamics to underlying archaeological phenomena.

The three selected regions in Central Europe have not yet been the subject of comparative studies of ^14^C sum calibrations, and their population dynamics have not yet been empirically correlated with local climate records. The regions are only separated by a few 100 km, so that small-scale interactions and dynamics can be detected. Furthermore, for our study regions, we hypothesize that climate variability and population dynamics limited access to resources, pushed resilience strategies, influenced the social organization system and had an impact on the degree of social inequality within the society.

## The study areas

The three selected regions, located in the low mountain and pre-Alpine ranges (Fig 1), show different landscapes and topographies with lake shores, plains and hills, which also shape the local climatic conditions.

The regions show divergent cultural developments and small-scale cultural phenomena for the Late Neolithic, respectively. In the Lake Constance area, pile-dwelling settlements of the Altheim, Pfyn and Horgen groups have been recorded. In the Danube region, mineral soil settlements with smaller houses and earthworks, e.g. of the Altheim and later Goldberg III and Chamer groups, have been documented (cf. Matuschik and Schlichtherle [41]). In the Circumharz region, the first copper-processing societies emerged with the Salzmünde/Baalberge groups and later with the Walternienburg/Bernburg group, which also established fortified hilltop settlements (cf. [42]. In the Czech Republic/Lower Austria region, the Baden Culture with the Boleráz group and from about 5000 calBP (3050 BCE) the Řivnáč Culture developed, while in Moravia, the Jevišovice group developed. The aforementioned Late Neolithic cultural groups were interconnected with varying intensity and showed linkages to the supraregional Globular Amphora phenomenon (cf. [43–45]. The Late Neolithic groups show a heterogeneous burial culture with megalithic graves and collective burials as well as single grave burials, cremations and inhumations (cf. [42, 45].

In contrast, the Corded Ware Phenomenon, the Bell Beaker Phenomenon, and the Early Bronze Age Únětice Society are widely interconnected socio-cultural phenomena. These groups are characterized by gender-specific single burials, sometimes with barrows, and small settlements with longhouses (cf. [46–48].

The Circumharz region is located in Central Germany, and the sites encompass the Harz low mountain range in the east, which reaches its highest elevation of 1,141 m on Mount Brocken. Sites are located primarily along the Saale River and its tributaries, the Unstrut and Bode Rivers, in the Thuringian Basin south of the Harz Mountains, in the Magdeburger Börde region north of the Harz Mountains, and in the Leipziger Tieflandbucht region east of the Saale River. In addition, most of the sites are located on the loess belt (Fig 1).

Freely exposed to westerly Atlantic winds, the western side of the Harz receives up to 1,600 mm of precipitation per year, while the eastern side, typical of the mountains, receives an average of only 600 mm of precipitation per year. The Circumharz lowlands are a very dry region with an annual precipitation of 450–475 mm (mean 1961–1990 CE) [49]. Winter temperatures range from 0.1 to 2 degrees on a long-term average; summer temperatures range from 16.1 to 18 degrees (average 1961–1990 CE) [49]. The loess soil in combination with the low precipitation is the basis for the prevailing fertile black earth soils in this area (cf. [50].

The second region includes the present-day Czech Republic and parts of the adjacent Austrian province of Lower Austria. The study area is naturally bounded by the Erzgebirge Mountains in the northwest, the Riesengebirge or Sudeten Mountains in the northeast, the Bohemian Forest in the southwest, and the Beskid Mountains in the east. Find concentrations are mainly located along the Elbe River and the Vltava River in the Bohemian Basin, and in the east of this study area along the Morova River, the Dyje River, and the Danube in Moravia. The find concentrations are particularly interrupted by the Bohemian-Moravian Uplands in the center of the Czech Republic. The sites correlate well with loess soils or loess derivatives in the study area (Fig 1), which is particularly evident in the Bohemian Basin and on the Moravian lowlands. The present annual precipitation in the basin landscapes fluctuates in the range from 400–700 mm p.a. (mean 1961–1990 CE). Winter temperatures in Prague, Central Bohemia, range from -1.9 to -0.6 degrees on a long-term average and summer temperatures range from 16.1 to 17.5 degrees; or in Brno, Moravia, winter: -2.5 to -0.6 degrees; summer: 17.0 to 18.5 degrees [51].

The third region is located in the Northern Alpine Foreland, mostly in Southern Germany, and includes Lake Constance, the Danube region, and its tributaries Inn, Isa, Lech, and Iller to the south. A concentration of sites is located at Lake Constance with a number of lakeside settlements. In addition to the lakeside settlements, the “Gäuboden” on the Danube, west of the Isar, and the Lechtal and Federsee Valleys in Upper Swabia also reflect concentrations of sites. Sites east of the Black Forest, along the upper reaches of the Neckar River, have also been recorded. Today, the Northern Alpine Foreland has higher precipitation amounts compared to the Circumharz lowlands, which measure about 850 mm at Lake Constance and between 700–800 mm p.a. along the Danube, and increase rapidly with increasing proximity to the Alps (mean 1961–1990 CE) [49]. Winter temperatures in Constance (Lake Constance) range from 0 to 1.3 degrees on a long-term average (1961–1990); summer temperatures range from 16.3 to 18.5 degrees; or in Augsburg, Swabia, in winter -1.5 to -0.1 degrees; summer: 5.5 to 17.3 degrees. Except for the lakeside settlements, the sites correlate well with the distribution of loess soils south of the Danube and along the Neckar (Fig 1).

## Materials and methods

### SPDs from calibrated radiocarbon dates

John Rick first introduced the idea of using the abundance of archaeological radiocarbon dates as an indicator of human population booms and busts in 1987 [54]. This approach was based on the assumption that “more people = more sites = more ^14^C dates”.

The increasing availability of large collections of archaeological (especially anthropogenic) radiocarbon dates has pushed up sum calibration of ^14^C data as a proxy of demographic change or of human activities in a broader sense.

Studies using larger samples and calibrated data have followed [55], and the calculation of SPDs has benefited from a number of methodological advances that have addressed criticisms of the effects of the calibration curve [56–58], biases in the research objectives and intensity of radiocarbon dating between regions [13, 22, 33], biases in the original archaeological dataset [33, 59, 60], the de-trending of the effects of taphonomic loss [61, 62], and the statistical testing of spatiotemporal trends in the variation of SPDs from calibrated radiocarbon dates [8, 18, 25, 26, 60, 63–66].

However, given the remaining gaps in available data, we recognize the limitations of inferring demographic trends from SPDs and calibrated radiocarbon data alone, and our results should be considered preliminary.

For our study, the radiocarbon data were calibrated and modeled using version 1.4.2 of the R package rcarbon [67]. All analyses and figures in this paper are reproducible by providing the dataset and a script written in the R statistical programming language (S2 Data). SPDs of non-normalized calibrated radiocarbon dates were produced for a period from 5500 to 3500 calBP (3550–1550 BCE). However, we calibrated and summed the radiocarbon probability distribution of each dataset over a slightly broader time range from 5700 to 2500 calBP (3750–550 BCE) to avoid boundary effects. We reduced oversampling of specific chronological site phases (e.g., due to biases in research objectives) by binning uncalibrated radiocarbon dates from the same site [26]. This approach mitigates both spatial and temporal inhomogeneity in the radiocarbon data available for the study regions.

Following previous work [56] showing that normalized calibrated data emphasize narrow artificial peaks in SPDs by steepening portions of the radiocarbon calibration curve, creating unwanted artifacts (we used IntCal20 throughout; [68], we chose to sum non-normalized distributions (see applications in Bevan et al. [8]; Palmisano et al. [13, 64]; Roberts et al. [30, 69].

**Fig 2:**
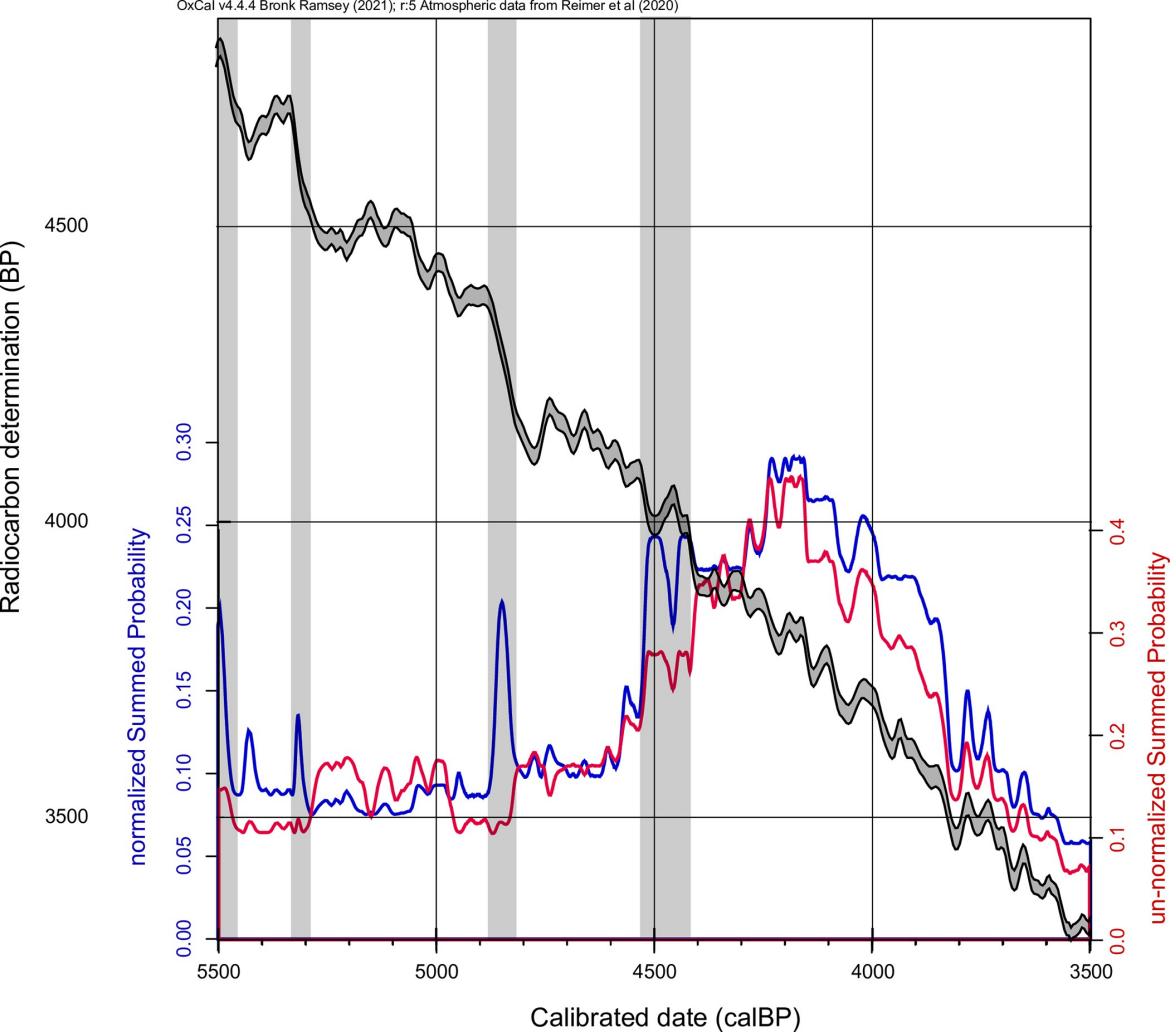
Comparison between normalized and non-normalized summed probability distributions of the Circumharz region with calibration curves. Gray vertical bands highlight artifacts where the curves differ.

The example from the Circumharz region clearly shows the difference between using normalized and non-normalized data. The normalized data clearly shows the peaks (gray bars) due to the steep parts of the calibration curve. These peaks are found especially in the Late Neolithic, e.g. at 4900–4800 calBP (2950–2850 BCE), and also in the Final Neolithic around 4500 calBP (2550 BCE) (Fig 2).

To test whether the inferred demographic trends described meaningful patterns that did not arise by chance, we compared the observed SPDs of the calibrated radiocarbon data to a theoretical null model of demographic change (Figs 5–7). This involved fitting a logistic growth model to the observed SPDs and calibrating random samples drawn from the fitted model (cf. Bevan and Crema [67] Shennan et al. [23], Timpson et al. [26]).

The resulting radiocarbon data were calibrated and their probability distributions summed to produce an expected SPD from the fitted model. This process was repeated 100 times to obtain a 95% confidence interval. Deviations above or below the 95% confidence limit of the envelope indicate periods of population growth (in red) or decline (in blue) greater than would be expected from a logistic model of population growth. The model does not provide a truly realistic description of real population growth, but it is useful for a quantitative assessment of population fluctuations [70].

SPDs are often compared to assess regional differences in population trends [30, 34] or to determine whether the relative proportions of different dated materials change over time. Following the method described in Crema et al. [18], we used a permutation test (permTest) for this purpose to assess the extent to which population fluctuations in each subregion (Circumharz region, Northern Alpine Foreland, Czech Republic/Lower Austria) deviate from the supraregional trend [18, 67]. In this procedure, labels are shuffled to identify the region from which each bin originates, and 100 SPDs are generated, from which a 95% critical gray envelope is derived. Deviations above and below the 95% confidence limits indicate periods in which the population increase or decrease for each subregion is greater or less than the trend for all regions.

The permutation test is robust to interregional differences in research intensity (and thus sample size) because the comparison is based on the “shape” of the SPDs (i.e., the relative change in the summed probabilities within each region) rather than on differences in their absolute magnitudes.

An alternative approach to modeling cumulative probabilities is to generate bootstrapped composite kernel density estimates (cKDE; for a detailed explanation of the method, see Brown [59, 60].

This method consists of randomly selecting calendar dates from each calibrated date and generating a kernel density estimate with a user-defined bandwidth (in this case, the bandwidth is 100). The process is repeated several times, and the resulting set of cKDEs is visualized as an envelope [67].

If the confidence interval is narrow, it is very likely that the observed pattern is a good representation of reality. This approach has the advantage of smoothing out calibration noise from SPDs and modeling uncertainty due to biases in the original input data (e.g., oversampling of certain chronological phases within the same archaeological site).

Overall, we are aware of the limitations of using radiocarbon data as a mere proxy for modeling demographic variation and will keep this in mind in subsequent interpretations in this paper.

Here, we use a total of 3426 radiocarbon dates from 589 sites, either from online sources and existing databases (e.g., RADON [71], EUROEVOL [72], EUBAR [73], CALPAL [74], p3k14c [75]), or from a variety of publications such as published reports, journal articles, etc. Particularly noteworthy here are the compilations in Tkáč and Kolář [14] for the Czech Republic and in Meller and Friederich [47] for the Circumharz region.

A total of 1521 ^14^C-dates from 147 sites from the Circumharz region, 961 ^14^C-dates from 261 sites from the Czech Republic/Lower Austria region and 944 ^14^C-dates from 181 sites from the Northern Alpine Foreland region were integrated (S1 Data). It is remarkable that within the smallest study region (Circumharz) the largest number of ^14^C data was collected and the highest clustering of data occurs here; partly with more than 450 data from a smaller find landscape (cf. Meller and Friederich [47] (Fig 1). This is mainly due to the numerous infrastructural construction projects that started in the Circumharz region after the German reunification, which led to numerous archaeological excavations and the generous use of radiocarbon dating.

^14^C dates with a standard deviation of 200 years or more were excluded because of their inaccuracy. These are mostly old dating from the 20th century. The total number of dates exceeds the suggested minimum threshold of n = 200–500 to produce reliable SPDs of calibrated radiocarbon dates with low statistical variation for a time interval [57] pp. 580–581).

As a relatively large number of ^14^C data are documented for each region, the dataset is robust to the effects of newly acquired data (also from a large project). In addition, the binning tool within the R script compensates for the effect of a larger number of ^14^C data extracted from a focused period and site. However, some possible research-induced biases are listed below and should be considered.

The SPD curve for the Circumharz region may be distorted by the ongoing focus of Early Bronze Age research triggered by the ’princely burials’ and the Nebra Sky Disk. At the same time, many Corded Ware and Bell Beaker burials were discovered, and chronological questions about these burials led to numerous 14C dates [76]. Most of the burials, however, were not found as a result of targeted research, but in connection with the construction of gas pipelines, railway lines and roads.

SPD biases for the area of the Czech Republic may be due to a strong research focus on Early Bronze Age hilltop settlements and trade routes associated with amber finds [77, 78].

In the northern Alpine foreland, intensive research on pile dwellings, which are UNESCO cultural heritage sites and have been more extensively researched than in the hinterland, may have led to a biased SPD result. Research into the Early Bronze Age around Singen and in the Lech Valley has also received increasing attention [37, 79, 80] and could also have biased the SPD curve. As a lot of data has already been collected, it is not expected that new data will lead to significant changes within the southwest German Early Bronze Age curve between 2200 and 1650 BCE.

Most of the data from the Circumharz region are from short-lived material such as bone. Furthermore, most of the data come from burial contexts, followed by settlement contexts. In the Czech Republic/Lower Austria region, a large proportion of the material is indeterminate. The second largest proportion of data comes from bone. The largest proportion is from a funerary context. However, the difference to the settlement context is not as large as in the Circumharz area. Moreover, in the Northern Alpine Foreland region, the predominant part also comes from bones, closely followed by wood and charcoal. However, the source situation is different compared to the other regions, since a larger proportion originates from settlement contexts. In summary, the data sources reveal different archaeological settings and research emphases in the regions. In the Circumharz region, graves are the predominant source genre. These have been uncovered especially in connection with route excavations. Settlements, especially in the Late Neolithic context, are disproportionately rare. In contrast, the Northern Alpine Foreland and the Czech Republic/Lower Austria regions show a more balanced pattern of finds, which is due to the large number of lakeside settlements in the Northern Alpine Foreland and the large amount of data from hilltop settlements in the Czech Republic/Lower Austria region (cf. S1 Data and Table 1).

**Table 1:** Regional comparison of ^14^C data source materials and sources of findings, each in percent.

Study regions/ material and arch. source (%)	bone	wood	charcoal	indeterminate	settlement	grave	indeterminate
Circumharz region	84	2	9	5	19	64	17
Czech Republic/Lower Austria region	35	4	16	45	25	34	41
Northern Alpine Foreland	31	24	17	28	33	29	38

### Palaeoclimate records

Disentangling the complex interactions between humans and the environment in empirical research approaches is a challenging task that requires quantitative reconstructions of different archaeological and palaeoenvironmental variables from these very different archives on finely tuned time scales. Regarding climate variability, numerous studies have demonstrated that hydroclimatic differences existed within Central Europe during the Holocene [81, 82]. However, because each proxy is shaped by its original local context through the interaction of various factors, interpreting hydroclimatic conditions from the selected isotopic signatures (δ18O, δ18O) of palaeoclimate records is rarely straightforward. For example, the isotopic signatures of Holocene speleothems do not necessarily reflect only humid or arid climates. More recently, speleothems have become important climate archives because they record climate variability (preferably in their O isotopic composition) at very high resolution and continuously, often even at decadal to annual resolution, and can be radiometrically dated very accurately and independently of ^14^C using U-series methods (for reviews see [83–85].

Central Europe, in contrast to Scandinavia and with the exception of the Alpine regions, has surprisingly few high-resolution climate archives, and quantitative precipitation reconstructions are lacking in particular. This is partially due to the circumstance that pollen records, a main source of terrestrial climate reconstructions, in central Europe are considered to be substantially biased by human activities and thus less reliable.

For our study regions, we have integrated palaeoclimate datasets from three sites in close proximity to our archaeological archives. Climate data in the form of oxygen isotope data of speleothems from the Spannagel Cave COMNISPA II [86], the Bleßberg Cave BB–1 [87], and the Bunker Cave [88] are added to allow us to investigate a causal relationship between demographic trends and climate variability. The O isotopic composition of the Bleßberg, Bunker, and Spannagel caves is considered a direct proxy for changes in atmospheric circulation and locally reflects the δ18O signature of precipitation over these caves [87].

Because present-day cave temperatures are in most cases consistently slightly above freezing and calcite deposition was continuous, temperature changes in speleothems during the Holocene were likely small. Therefore, the δ18O signal from calcite is considered to largely reflect atmospheric δ18O changes [86].

Some authors have suggested that the δ18O of speleothem calcite represents the amount of precipitation during the winter recharge period, and thus higher (lower) values indicate drier (wetter) seasonal conditions during winter rather than average annual conditions [89–91].

#### Spannagel Cave COMNISPA II

Spannagel Cave is located in the rear part of the Tux Valley (Zillertal Alps, Western Austria, Lat.: 47.080; Lon.: 11.671). The cave extends over a length of 11 km from 2524 to 2198 m asl and was formed in a contact karst landscape.

COMNISPA II is an example of a combined stable isotope record [86, 92] that has been used to interpret Holocene climate variability [93]. This dataset is based on composite data from five stalagmites (SPA 12, 70, 127 and 128) from Spannagel Cave (Source: https://www.ncei.noaa.gov/access/paleo-search/study/14510), which are characterized by clean, U-rich calcite that allows precise U/Th chronology.

The δ18O values for Spannagel Cave are interpreted by Fohlmeister as higher values indicating a colder climate and vice versa [86] p. 753). The relationship between temperature and δ18O in Spannagel Cave can be explained by the different contributions of summer and winter precipitation to the karst reservoir that feeds the drip sites. This change in precipitation pattern, especially in winter, is closely related to temperature changes [94].

Several studies have shown that the COMNISPA records are representative of the alpine climate [92, 95, 96]. However, other studies have also shown that the observed O signal from this site follows large-scale atmospheric climate variations. For example, Spannagel’s δ18O records and growth phases agree quite well with the δ18O pattern in Greenland ice cores on millennial to orbital time scales [97, 98], with sedimentary proxies and deep-sea coral growth from the North Atlantic [99, 100], and with other stalagmites from Central Europe [93].

#### Bleßberg Cave (BB-1)

The NW-SE oriented Bleßberg Cave is located at Lat.: 50.424 and Lon: 11.020 at about 500 m above sea level on the southern edge of the Thuringian Slate Mountains. The cave had no natural entrance before its discovery, but is drained by a small stream, and the cave is covered by 12 to 50 m (35–40 m above the sampling site) of marly limestone (Lower and Middle Muschelkalk).

Samples of two stalagmites are available for Bleßberg Cave. Stalagmite BB-1 grew between 5.6 and 0.6 calBP (kiloyears before present, i.e. 1950 AD), while BB-3 grew between 11.9 and 5.4 calBP. For our study, we used sample BB-1 because of its temporal coverage.

The δ18O record of Bleßberg Cave (δ18Ospel) integrates precipitation history and the corresponding δ18O signal in precipitation (δ18Op), seasonal changes in infiltration, and temperature in a complex manner, capturing longer-term, trans-regional environmental dynamics. Given the observed seasonal infiltration pattern, both dripwater and speleothem δ18O values represent an average seasonal signal that is slightly biased towards the winter season.

Lower δ18O values in Bleßberg Cave indicate colder and drier conditions under a pronounced Siberian high and a negative North Atlantic Oscillation (NAO) influence, whereas wetter and warmer (maritime) conditions reflect higher δ18O values with a positive NAO influence [87].

#### Bunker Cave

Bunker Cave is located at Lat.: 51.22003 and Lon: 7.39053 in Sauerland, West Germany, and is part of a large cave system consisting of several caves close to each other. The cave entrance is located at 184 m above sea level on a south-facing slope. Four speleothems were sampled that grew within 12 m of each other (Bu1, Bu2, Bu4, and Bu6) (Fohlmeister et al. 2012, p. 1752) (Source: https://www.ncdc.noaa.gov/paleo/study/20589).

Winter precipitation accounts for most of the cave drip water and dominates its δ18O value. The relationship between surface air temperature and precipitation δ18O is positive (_ 0.1–0.3‰_C-1). Fohlmeister and colleagues interpret the observed variations in cave δ18O as changes in winter surface temperature and winter precipitation. More positive δ18O values reflect cold and dry winters, while more negative δ18O values represent warmer and wetter winters. As pointed out in Langebroek et al. [101], the correlation pattern between the δ18O precipitation value and the atmospheric circulation over Europe is the result of the combined effect of temperature and precipitation. Heat and moisture are mainly transported to the European continent by westerly winds from the North Atlantic. Therefore, climate-related signals from the North Atlantic (e.g., the hematite-stained grain (HSG) record in marine sediments; [81] and the δ18O record from Bunker Cave are expected to show similar variations [88] p. 1759).

Throughout the Holocene, the composite δ18O record of the four Bunker Cave stalagmites shows values between -7 and -5‰. Comparing the δ18O value of Bunker Cave with δ18O values from Atta Cave in Western Germany [102, 103], Katerloch in Southern Austria [104], and Spannagel Cave [92], all records show a similar structure. This suggests that the signal encoded in the stalagmites of Bunker Cave represents transregional climate variability. Comparison of the composite record from Bunker Cave and the other European stalagmite archives with the HSG record from the North Atlantic [81] suggests that the signal from the Central European continent may even be representative of the North Atlantic region and much of Europe [88], p. 1760).

## Results

### Demographic trends

The SPD of the non-normalized calibrated radiocarbon dates from the Circumharz area (Figs 4, 5) begins around 5500 calBP (3550 BCE) with the Salzmünde and Baalberge groups, whose occurrence is associated with a significant positive deviation from the null model. The cKDE and SPD show a moderate increase in data around 5350 calBP (3400 BCE). This continues to be associated with the Salzmünde group and the incoming Walternienburg/Bernburg groups. In the later course of the Late Neolithic, a population decline occurs between 5000 (3050 BCE) and 4900 calBP (2950 BCE), which results in a negative deviation of the population size from the null model until 4700 calBP (2750 BCE).

In the Final Neolithic, from 4900 calBP (2950 BCE), we see a sharp increase in SPD, which is accompanied by the appearance of the Globular Amphora Culture, the Corded Ware phenomenon, and from 4450 calBP (2500 BCE), the onset of the Bell Beaker phenomenon, leading to a positive deviation from the null model that extends into the Early Bronze Age around 4000 calBP (2050 BCE). From 4200 calBP (2250 BCE), a transitional phase in material culture from the Corded Ware and Bell Beaker phenomena to the material culture of the Únětice group is documented, which appears as a continuous process (cf. Großmann [46], p. 251; Schwarz [105], p. 703).

**Fig 3:**
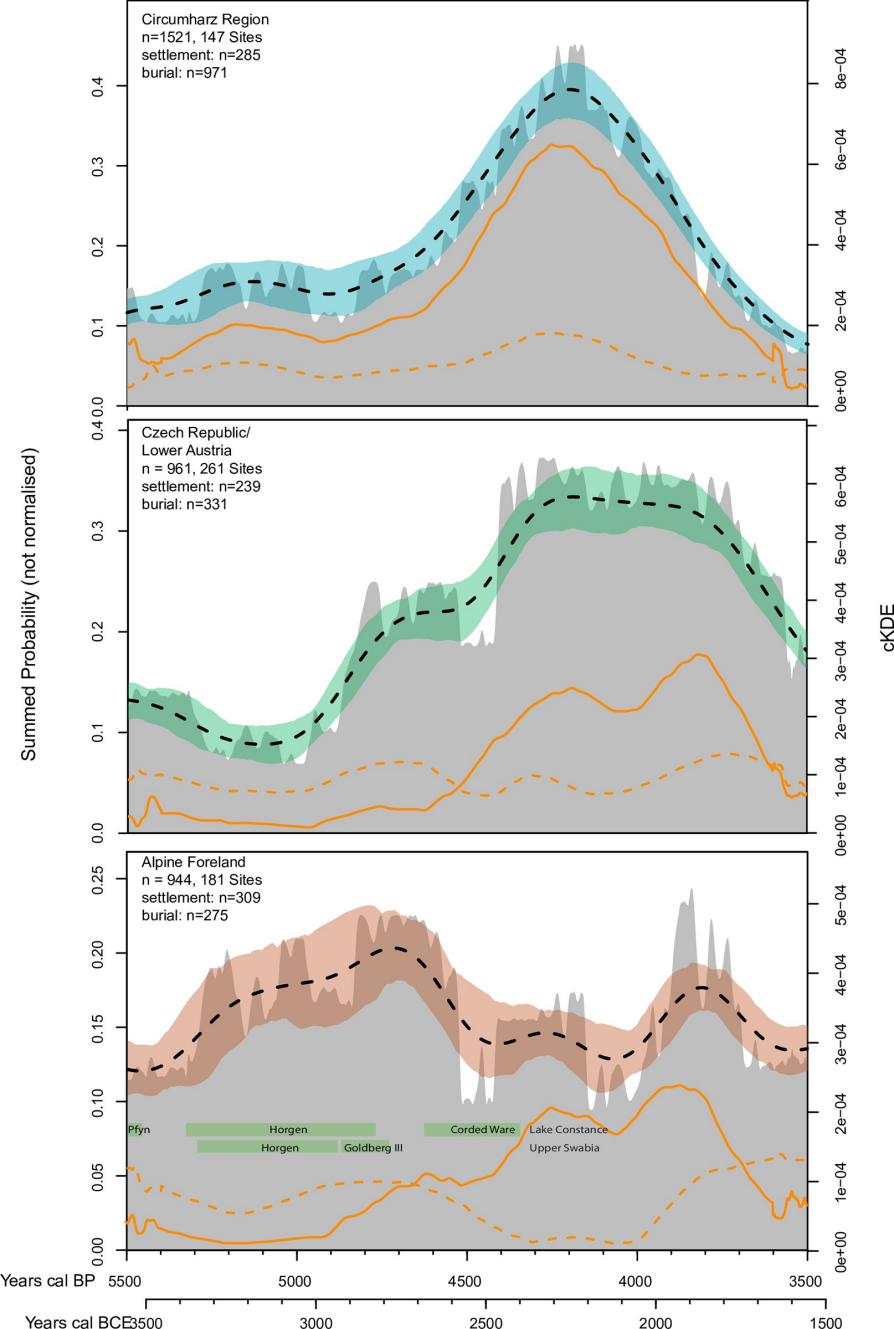
Summed probability distributions (SPD) and composite kernel density estimations (cKDE). SPD of non-normalized calibrated radiocarbon data (gray), solid orange SPD median lines for burials, and dashed lines for settlements. Bootstrapped composite kernel density estimation (cKDE) of non-normalized calibrated radiocarbon data (with colored deviation bands, and dashed median lines). In addition, the pre-alpine lakeshore settlement phases have been plotted (cf. Haack [107]).

**Fig 4:**
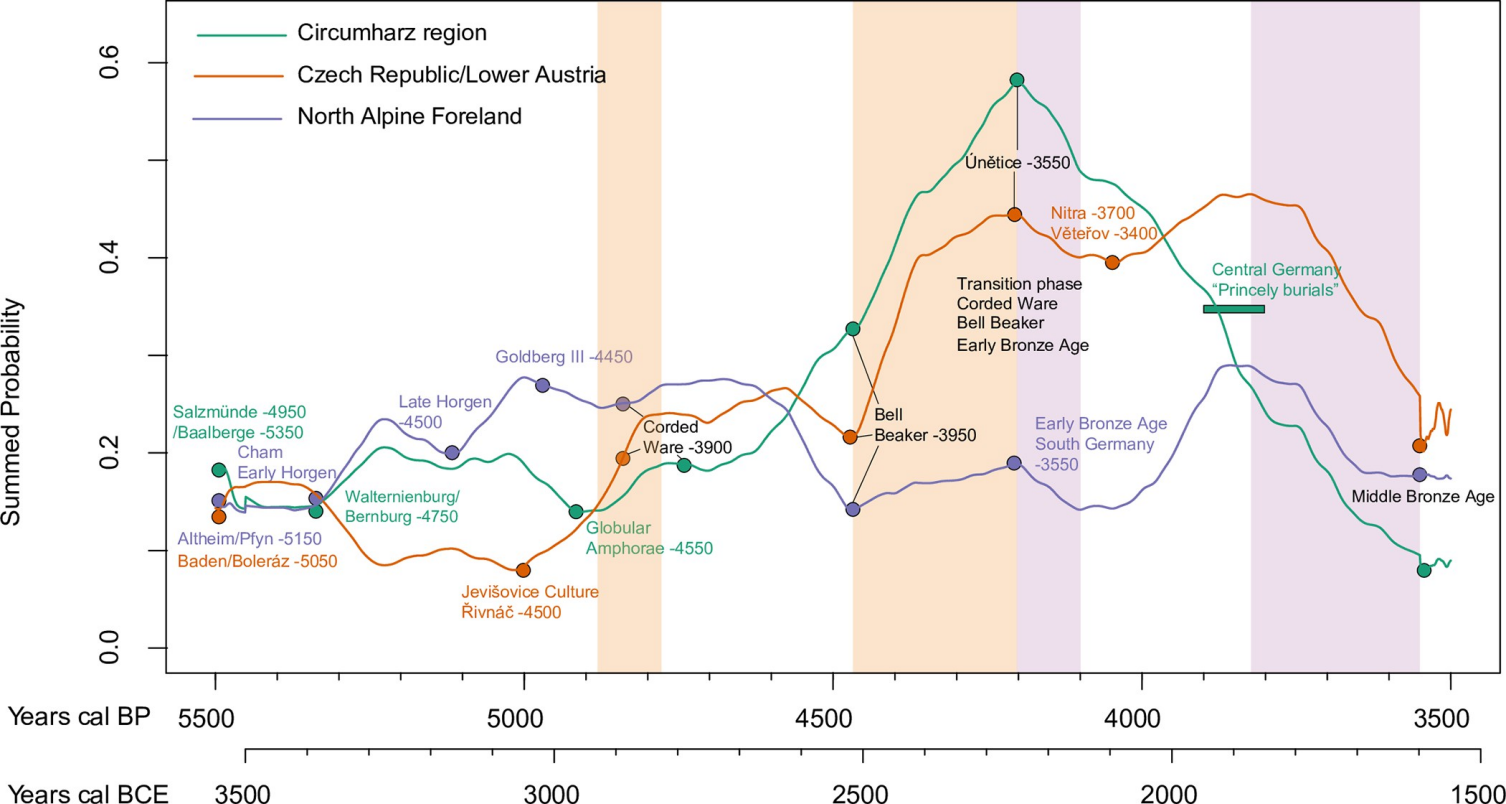
Comparison of the SPD median curves of the regions. Inserted are starting points of archaeological groups as well as the time (in BP) when they dissolve. Vertical light orange bands = boom phases across regions; vertical light purple bands = bust phases across regions.

**Fig 5:**
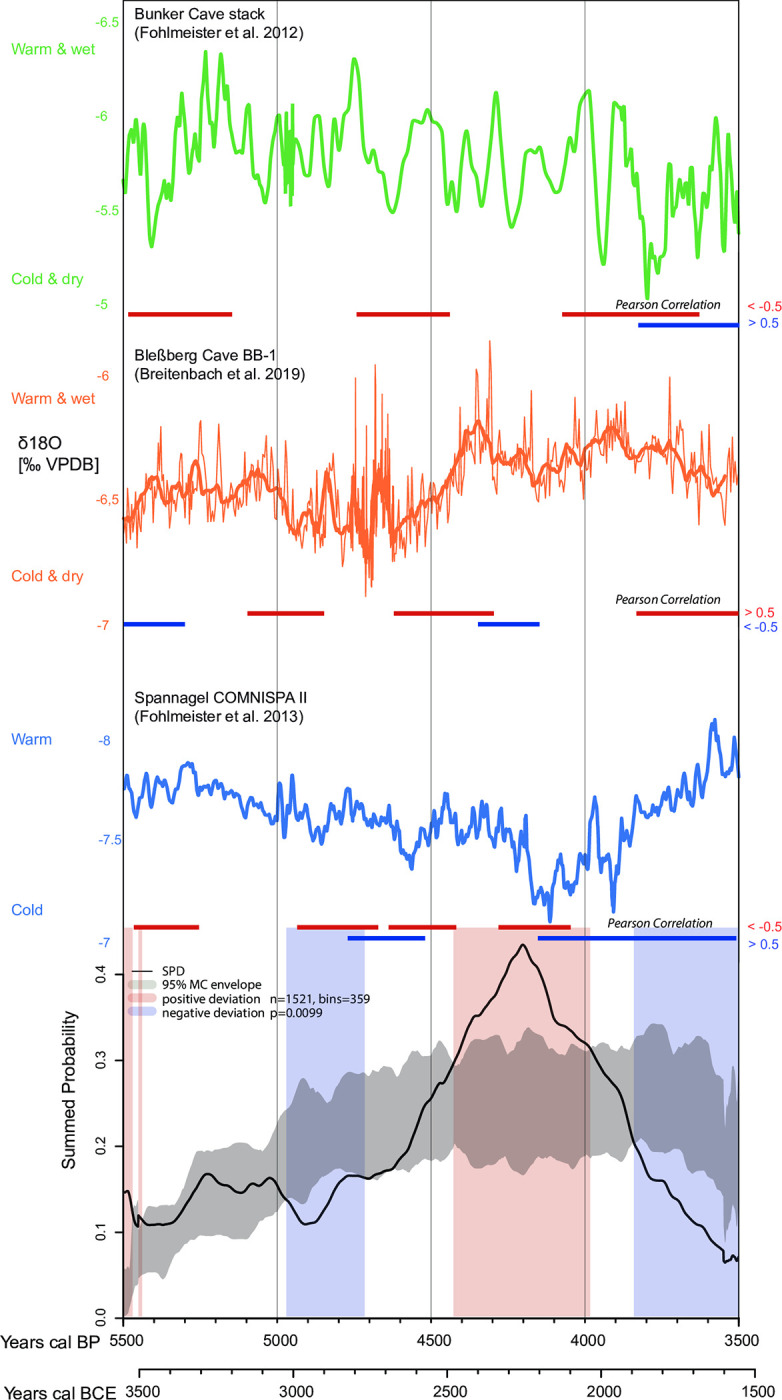
Circumharz region. Summed probability distribution (SPD) of non-normalized calibrated radiocarbon data compared to a fitted logistic model (gray envelope with 95% confidence). The light blue and light red vertical bands indicate the chronological regions within the observed SPD that deviate negatively and positively from the null model, respectively. The image is supplemented with δ18O-climate curves from the speleothems from Bunker Cave, Bleßberg Cave, and Spannagel Cave. Phases of Pearson linear (r) correlations above +0.5, below -0.5 and p-value <0.025 are plotted as red and blue horizontal lines, respectively.

From the transition phase from the Final Neolithic to the Early Bronze Age (4200 calBP/2250 BCE), there is a continuous decline of the population, which leads to a significant and negative deviation from the logistic null model from 3850 calBP (1900 BCE). The decline continues until the end of the study period (3500 calBP/1550 BCE) or until the Middle Bronze Age (Figs 3–5).

**Fig 6:**
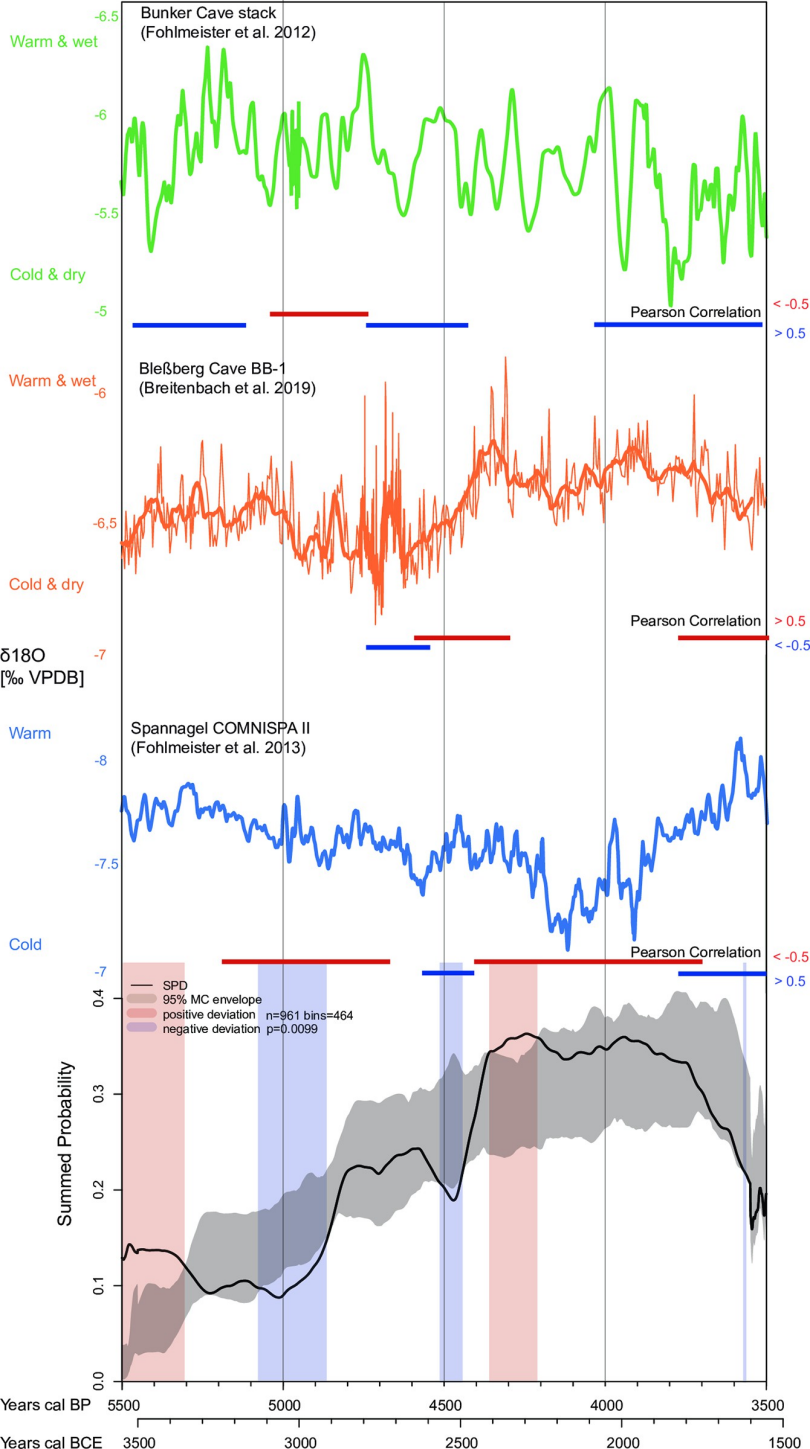
Czech Republic/Lower Austria region. Summed probability distribution (SPD) of non-normalized calibrated radiocarbon data compared to a fitted logistic model (gray envelope with 95% confidence). The light blue and light red vertical bands indicate the chronological regions within the observed SPD that deviate negatively and positively from the null model, respectively. The image is supplemented with δ18O-climate curves from the speleothems from Bunker, Bleßberg, and Spannagel caves. Phases of Pearson linear (r) correlations above +0.5, below -0.5 and p-value <0.025 are plotted as red and blue horizontal lines, respectively.

In the Czech Republic/Lower Austria region (Figs 3, 4, and 6), there is a positive deviation from the null model between 5500 (3550 BCE) and 5350 calBP (3400 BCE) when the Baden/Boleráz group is formed. From 5300 calBP (3350 BCE), a decrease in the SPD for the Late Neolithic is noticeable, leading to a significant negative deviation from the null model between 5100 calBP and 4850 calBP (3150–2900 BCE). From 5000 calBP (3050 BCE), a steady increase of the SPD begins, which is first associated with the Jevišovice and Řivnáč groups, and from 4850 calBP (2900 BCE) with the Corded Ware phenomenon. Around 4600 calBP (2650 BCE), a decrease in the SPD begins, which results in a negative deviation from the null model between 4500 calBP and 4450 calBP (2550–2500 BCE). Beginning at 4450 calBP (2500 BCE), a sharp increase in the SPD begins with the onset of the Bell Beaker phenomenon, resulting in a positive deviation from the null model between 4350 and 4200 calBP (2400–2250 BCE). Similar to the Circumharz region, a transitional phase from the Corded Ware/Bell Beaker phenomenon to the Únětice group is evident around 4200 calBP (2250 BCE). Looking at the SPD median line, we see a small population dip between 4200 and 4000 calBP (2250–2050 BCE) and from 4000 calBP (2050 BCE). Accompanied by the appearance of the Nitra and Věteřov groups, we observe an increase in population density. Another peak of population density is reached in 3850 calBP (1900 BCE). Thereafter, a continuous decrease in the SPD begins, which further intensifies around 3750 calBP (1800 BCE) and undergoes a short significant deviation around 3550 calBP (1600 BCE) (Figs 3, 4, and 6).

**Fig 7:**
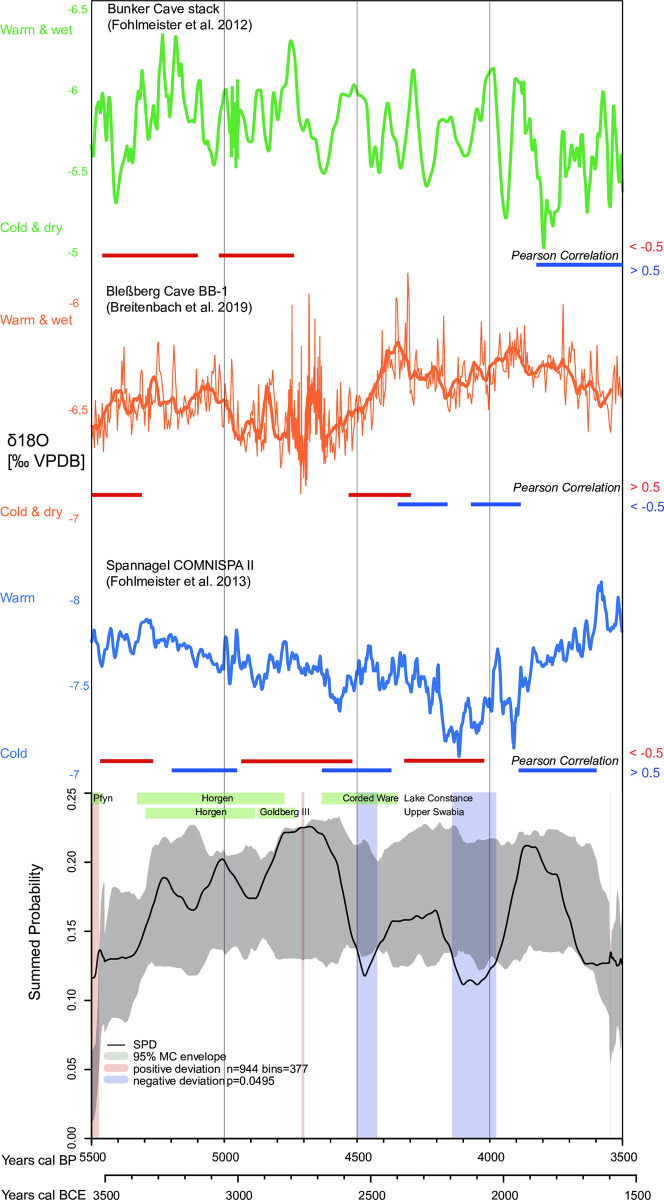
Northern Alpine Foreland. Summed probability distribution (SPD) of non-normalized calibrated radiocarbon data compared to a fitted logistic model (gray envelope with 95% confidence). The light blue and light red vertical bands indicate the chronological regions within the observed SPD that deviate negatively and positively from the null model, respectively. The image is supplemented with δ18O-climate curves from the speleothems from Bunker, Bleßberg, and Spannagel caves. Phases of Pearson linear (r) correlations above +0.5, below -0.5 and p-value <0.025 are plotted as red and blue horizontal lines, respectively. Additionally, the pre-alpine lakeshore settlement phases have been plotted (cf. Haack [107]).

In the Northern Alpine Foreland (Figs 3, 4, and 7), a brief positive deviation from the null model is observed around 5500 calBP (3550 BCE). The SPDs show boom phases around 5350 calBP (3400 BCE) and 5100 calBP (3150 BCE), which are followed by rapidly declining phases. This is due to the appearance and disappearance of lakeside settlements of the Horgen Culture group. With the appearance of the Goldberg III group around 4950 calBP (3000 BCE) and the beginning of the Corded Ware phenomenon around 4850 calBP (2900 BCE), the population size stabilizes at a comparatively high level, which leads to a significant positive deviation from the null model around 4700 calBP (2750 BCE).

From 4650 calBP (2700 BCE) onward, a decline in the SPD is also evident, due to the postulated decrease in lakeshore settlement, leading to a negative deviation from the logistic null model at 4400 calBP (2450 BCE). The reason for this is most likely a higher lake level due to a colder climate [106]. This negative deviation may represent a biased source-related shift rather than a demographic one.

The Bell Beaker phenomenon shows a slight increase in SPD from 4450 calBP (2500 BCE) onwards. Similar to the Czech Republic/Lower Austria region, we see a collapse of population density between 4200 and 4050 calBP (2100 BCE) at the transition to the Early Bronze Age, which leads to a negative deviation of population size between 4150 and 4000 calBP (2200–2050 BCE). At 4000 calBP (2150 BCE), a boom begins with the Southern German groups of the Early Bronze Age (Singen group, Straubing group, Lech group, cf. Massy [80], which, similar to the Czech Republic/Lower Austria region, reaches its highest peak around 3850 calBP (1900 BCE). From this point until the beginning of the Middle Bronze Age (3550 calBP/1600 BCE), a decrease of the sum-calibrated dates can be observed, similar to the other study regions (Figs 3, 4, and 7).

Based on the comparison of the SPDs from Fig 4 and the logistic models (Figs 5–7), common boom and bust phases of the regions can be summarized. Common boom phases are shown in the Late Neolithic around 5500 calBP (3550 BCE) with the Late Neolithic groups Salzmünde/Baalberge, Altheim/Pfyn, and the Boleráz group of the Baden Society, and in the Final Neolithic with the appearance of the Globular Amphora phenomenon and the Corded Ware phenomenon between 4900 and 4800 calBP (2950–2850 BCE) and between 4450 and 4200 calBP (2500–2250 BCE) when the Bell Beaker Phenomenon caused a population boom in addition to the Corded Ware Phenomenon. Shorter, parallel phases of decline in individual regions are shown around 5000 calBP (3000 BCE) with the dissolution of Late Neolithic societies, around 4600 calBP (2650 BCE) with the population decrease of the Corded Ware Phenomenon (not in the Circumharz area), and between 4200 and 4000 calBP (2250–2050 BCE) in the transition phase to the Early Bronze Age. From about 3850 calBP (1900 BCE), a general decrease of the population can be verified in all regions until the Middle Bronze Age (3550 calBP/1600 BCE).

### Comparison of settlement and burial dates

In addition to the total number of ^14^C dates, the main sources of ^14^C dates were analyzed separately to identify possible biases (Fig 3). These include ^14^C data from settlement and burial contexts. Differences in pattern and intensity are clearly visible. In the Czech Republic/Lower Austria region and in the Northern Alpine Foreland, significantly more ^14^C dates for the Late Neolithic are documented in settlement contexts than in grave contexts. This becomes clear, for example, in connection with the lakeshore settlements in the Lake Constance region, where we have relatively many documented and absolutely dated houses, but hardly any burials. From the Final Neolithic onwards, the gap between data from burial and settlement contexts widens. We see a jump in burial data in all regions. On the one hand, this is due to the surplus of documented burials, however, on the other hand, relatively few houses have been absolutely dated (this is especially true for the Northern Alpine Foreland region), since reliable material for absolute dating is much rarer in mineral soil settlements than in burial contexts.

The Pearson correlations show that there are significant correlations between the ^14^C sources in all regions. This means that there is a systematic correlation between the settlement and burial curves. In the Circumharz region, this correlation is strongly positive, so that large distortions can be largely excluded. In the Czech Republic/Lower Austria region, there is a weak positive correlation. Accordingly, the settlement and grave curves run parallel, but the settlement data are slightly overrepresented in the Late Neolithic and slightly underrepresented in the Final Neolithic/Early Bronze Age (grave data vice versa). In the Northern Alpine Foreland, settlement and grave data run in opposite directions. The number of settlement dates is strongly overrepresented in the Late Neolithic and strongly underrepresented in the Final Neolithic/Early Bronze Age (Table 2). This means that especially the Late Neolithic population dynamics described above have to be considered with caution due to a biased database, and this result has to be taken into account in the following analyses.

**Table 2:** Pearson correlation coefficient r-value matrix for the correlation between burials and settlements in the respective regions. Significant correlations are indicated by bold numbers. p < 0.01. Degree of correlation: small (> ± 0.10), moderate (> ± 0.30), large (> ± 0.50) according to Cohen and Cohen [108].

		Circumharz region	Czech Republic/Lower Austria region	Northern Alpine Foreland
		**Settlements**		
Circumharz region	**Graves**	**0.8290309**		
Czech Republic/Lower Austria region			**0.1536673**	
Northern Alpine Foreland				**-0.3157705**

### Comparison of population development between the regions

The following section compares population trends between the regions. Pairwise Pearson correlations between the SPDs are calculated to evaluate the demographic patterns between the regions.

The results for the whole study period show an almost strong and significant positive correlation between the Circumharz region and the Czech Republic/Lower Austria region. This indicates a generally parallel population development. All other correlations are slightly negative (Table 3).

**Table 3:** Pearson correlation coefficient r-value matrix for a correlation of population curves between regions from 5500–3500 calBP. Significant correlations are indicated by bold numbers. p < 0.01. Degree of correlation: small (> ± 0.10), moderate (> ± 0.30), large (> ± 0.50) according to Cohen and Cohen [108].

Linear (r) Pearson Correlation 5500–3500 calBP	Circumharz region	Czech Republic/ Lower Austria region	Northern Alpine Foreland
Circumharz region	1	**0.4771**	-0.14736
Czech Republic/Lower Austria region		1	-0.089467
Northern Alpine Foreland			1

In the next step, only the SPDs of the Late Neolithic and the beginning of the Final Neolithic (5500–4500 calBP, 3550–2550 BCE) as well as the Late Final Neolithic and the Early Bronze Age (4500–3500 calBP (2550–1550 BCE) are correlated. Looking at the SPD median curves, we note (Fig 4) a parallel trajectory between the Northern Alpine Foreland and Circumharz curves in the Late Neolithic. If we compare this thesis by means of a Pearson correlation test, it is confirmed. This becomes particularly clear between 5500 and 4800 calBP (3550–2850 BCE) up to the beginning of the Corded Ware phenomenon, where a significant coincidence is visible. The trajectory of the Czech Republic/Lower Austria region does not show any concordance with the other two regions in this phase (Table 4).

**Table 4:** Pearson correlation coefficient r-value matrix for a correlation of population curves between regions from 5500–4500 calBP. Significant correlations are indicated by bold numbers. p < 0.01. Degree of correlation: small (> ± 0.10), moderate (> ± 0.30), large (> ± 0.50) according to Cohen and Cohen [108].

Linear (r) Pearson Correlation 5500–4500 calBP	Circumharz region	Czech Republic/ Lower Austria region	Northern Alpine Foreland
Circumharz region	1	-0.060078	0.29723 **0.69356 (5500–4800 BP)**
Czech Republic/Lower Austria region		1	0.03159
Northern Alpine Foreland			1

From 4500 calBP (2550 BCE), we see an apparent parallel development between the Czech Republic/Lower Austria region and the Northern Alpine Foreland with the onset of the Bell Beaker phenomenon. Statistically, the synchrony shows up as a strong correlation in Pearson’s correlation test. The Circumharz region shows an almost moderate correlation with the Czech Republic/Lower Austria region. This can be explained by the dominant Únětice phenomenon in both regions (Table 5).

**Table 5:** Pearson correlation coefficient r-value matrix for a correlation of population curves between regions from 4500–3500 calBP. Significant correlations are indicated by bold numbers. p < 0.01. Degree of correlation: small (> ± 0.10), moderate (> ± 0.30), large (> ± 0.50) according to Cohen and Cohen [108].

Linear (r) Pearson Correlation 4500–3500 calBP	Circumharz region	Czech Republic/ Lower Austria region	Northern Alpine Foreland
Circumharz region	1	0.29274	-0.0023639
Czech Republic/Lower Austria region		1	**0.66655**
Northern Alpine Foreland			1

Overall, there is a synchronous population dynamic in the Late Neolithic for the Circumharz region and the Northern Alpine Foreland. Looking back at the different SPDs of the settlement and burial data from the Northern Alpine Foreland, we can assume a possibly realistic population dynamic for the Late Neolithic in the Northern Alpine Foreland on the basis of this synchrony. Within the Final Neolithic and the Early Bronze Age, a synchronous population dynamic between the Czech Republic/Lower Austria region and the Northern Alpine Foreland can be demonstrated. The Circumharz region, however, shows a divergent development.

### Permutation tests

**Fig 8:**
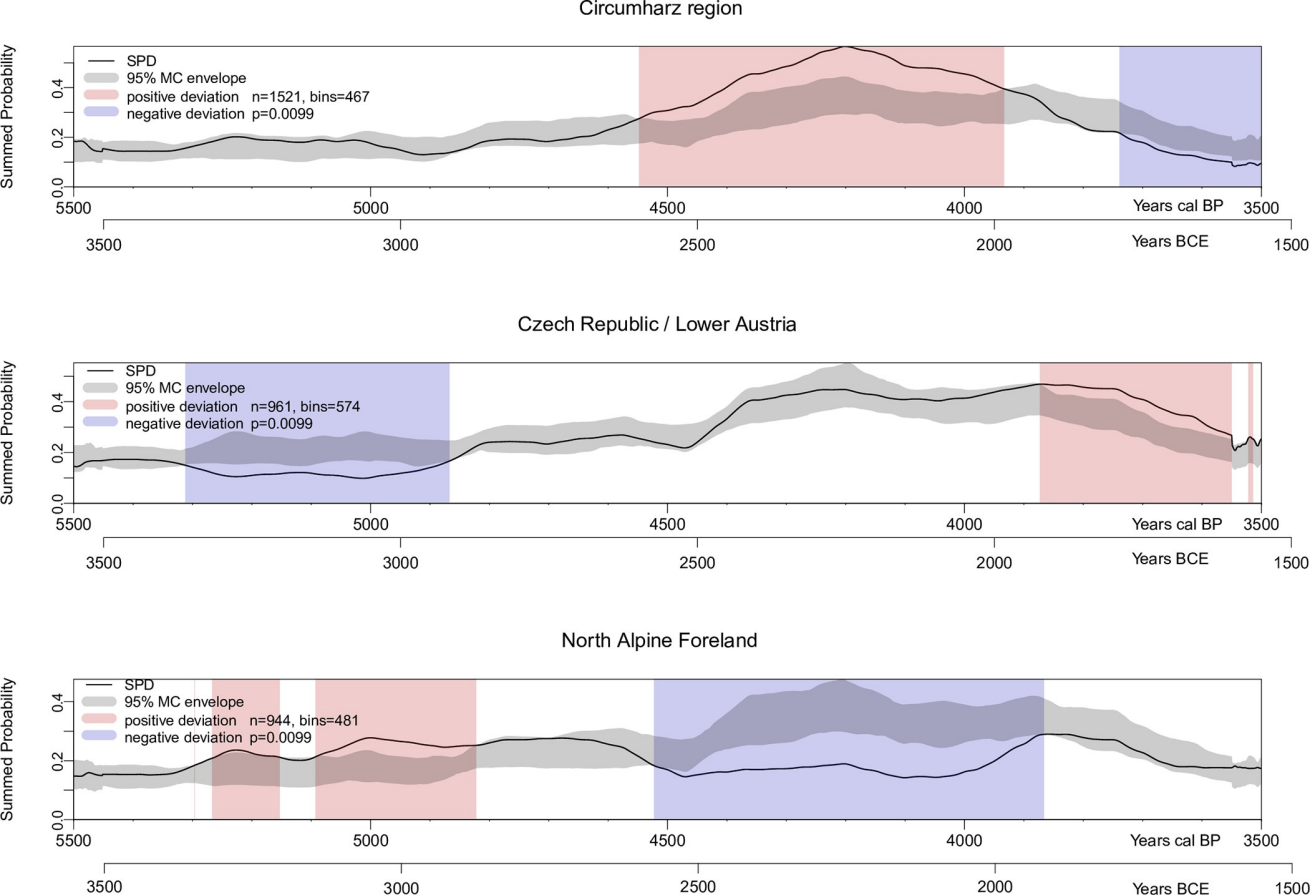
Permutation test comparing the three SPDs. The light blue and light red vertical bands indicate the chronological regions within the observed SPDs that deviate negatively and positively from the null model, respectively.

Fig 8 shows the regionally subdivided SPDs of the non-normalized radiocarbon data compared to the overall trend of all three regions (gray envelope) using a permutation test. Such a technique also addresses sample size issues, as the resulting gray envelopes of the pan-regional trend are larger in the subregions with less radiocarbon data, reflecting greater uncertainty. All three subregions show significant long-term deviations from the pan-regional trend (p-value < 0.01).

In the Circumharz region, the population density during the Final Neolithic and the older Early Bronze Age (~ 4600–3900 calBP, 2650–1950 BCE) is significantly above the supraregional pattern, while during the transition to the Late Early Bronze Age/Middle Bronze Age (~ 3800–3500 calBP, 1850–1550 BCE) there was a comparatively low population.

The Czech Republic/Lower Austria region shows a negative local variation during the Late Neolithic between 5300 and 4800 calBP (3350–2850 BCE), while the Late Early Bronze Age population (~ 3800–3600 calBP, 1850–1650 BCE) shows a higher population density compared to the other regions.

The Northern Alpine Foreland shows a positive deviation from the supraregional pattern within the Late Neolithic between ~ 5300–4800 calBP (3350–4850 BCE). In contrast, a significant decrease from the overall trend is observed during the Final Neolithic between 4600 and 3800 calBP (2650–1850 BCE). However, due to the overrepresentation of Late Neolithic settlement data, we must assume a slightly biased data set and view the SPD predominance with caution.

The permutation test shows that individual regions exhibit a population increase compared to the supraregional pattern, but at the same time other regions show a population decrease. What are the reasons for this?

The reason for the Late Neolithic population growth in the Northern Alpine Foreland could be a prosperous phase compared to neighboring regions, a distorted database due to the large amount of documented settlement data, and possibly a (partial) migration also from the neighboring Czech Republic/Lower Austria region, which could be indicated by pottery sherds of the Baden group in the Northern Alpine Foreland (cf. Szmyt [44]; Furholt [43]).

The significant population increase in the Final Neolithic in the Circumharz region may be related to several factors. One obvious cause, which is also supported by aDNA studies, could be a partial migration of the carriers of the Corded Ware phenomenon from the Baltic forest-steppe region, whereby especially the Early Corded Ware shows a high genetic diversity with a native substrate [109] Fig 4). Regarding the Bell Beaker phenomenon, genetic analyses show that it may have originated in the Rhine region and then migrated further east, including the Circumharz region [109] p. 7). A migration of Bell Beaker people from the Northern Alpine Foreland is possible, as suggested by the permutation test, based on aDNA data [109] and material similarities [46]. However, Furholt has rightly argued that such migration processes are complex and case-specific [110], and that there should be no unification of biological and archaeological categories [111].

In the Early Bronze Age, we observe a significant increase of the SPD in the Czech Republic/Lower Austria region, which may be primarily related to an internal prosperous economic development. At the same time, a significant decrease of the population in the Circumharz region can be proved. The Únětice group resided in both regions at that time. Therefore, a partial migration of the Únětice group from the Circumharz region to the study area in the Czech Republic/Lower Austria region or to other regions is conceivable. There is at least some evidence for gene flow from the Northeastern Europe/Baltic region to Bohemia, which correlates well with the emerging trade in amber from the Baltic [78, 109].

Whether the population decline in the Circumharz region was due to changing environmental conditions will be investigated in more detail below.

### Climatic evidence

Regional climatic variations are observed in the study areas during the Middle and Late Holocene. A HTM is however not consistently recorded in the three speleothem records considered to reflect mainly winter conditions. The speleothems from the Bleßberg, Spannagel, and Bunker caves suggest that from 5500 to 5400 calBP (3550–3450 BCE) and from 5300 to 5100 calBP (3350–3150 BCE) a predominantly warm and humid climate with mild winters prevailed in all regions. From about 5100 calBP (3150 BCE), the overall trend changed to a colder and drier climate after the end of the Holocene Thermal Maximum, which culminated in the 4.2 ky event. This is particularly evident in the δ18O signatures from Spannagel Cave (Fig 7). This phase is interrupted by several warmer and wetter phases, e.g. between 4850–4650 calBP (2900–2700 BCE) and 4550–4300 calBP (2600–2350 BCE). In particular, the Bleßberg record indicates an increasingly warm and humid climate. In the transition from the Middle Holocene to the Late Holocene, between about 4200 calBP and 4100 calBP (2250–2150 BCE), i.e. the phase of the so-called 4.2 event, all speleothems indicate a general cooling and a dry winter climate. From 4100 calBP to about 3900 calBP (2150–1950 BCE), all curves show a trend towards an increasingly warmer and wetter climate. While from 3900 calBP to 3500 calBP (1950–1550 BCE) the curve from the Alpine Spannagel Cave shows a trend towards a predominantly warm climate, the northern curves from the Bleßberg and Bunker caves from 3900 calBP (1950 BCE) onwards show a clear trend towards an increasingly cooler and drier climate (Figs 5–7). Breitenbach and colleagues [87] associate the δ18O values for the Bleßberg Cave from 3900 calBP (1950 BCE) onward with a moderate to strong influence of the Siberian High and a pronounced negative NAO. The lower δ18O values indicate cold winter conditions with low precipitation [87, 112].

### Comparison of demographic and climatic trends

When comparing the climate and population curves (SPDs), there are clear parallels in their trajectories.

The positive and negative significant deviations of the population trends shown in Figs 5–7 are well reflected in the climate curves. For example, the positive significant population deviations correlate very well with the warm/humid winter climate indicators of the Bleßberg Cave (high δ18O values) as well as with the warm/humid indicators of the Spannagel and Bunker Caves (low δ18O values in this case).

This is evident in all regions in the Late Neolithic between 5500 and 5400 calBP (3550–3450 BCE). For the Final Neolithic, between 4400 and 4200 calBP (2450–2250 BCE), significantly high population densities in the Circumharz region as well as in the Czech Republic/Lower Austria region correlate with warm and humid climate data. In the Northern Alpine Foreland, on the other hand, a warm climate positively correlates with population growth in the Early Bronze Age between 4000 and 3850 calBP (2050–1900 BCE), which is particularly evident from the data of the Northern Alpine Foreland burials.

Similarly, negative population deviations correlate significantly with cold and dry winter climate indicators of the Bleßberg Cave (low δ18O values) as well as with cold indicators of the Spannagel and Bunker Caves (in this case high δ18O values). This is shown, for example, in the Circumharz region and in the Czech Republic/Lower Austria region between about 5000 and 4900 calBP (3050–2950 BCE) as well as in the Czech Republic/Lower Austria region and in the Northern Alpine Foreland between 4600 and 4500 calBP (2650–2550 BCE), when the climatic proxies indicate a cool and dry climate. For the Northern Alpine Foreland, there is a significant population decline between 4200 and 4100 calBP (2250–2150 BCE), as indicated by the climate proxies. The nearby Spannagel Cave climate proxy shows the coldest climate of the entire study period during this phase. The significant population decline in the Circumharz region starting at 3850 calBP is particularly striking. This decrease correlates strongly with the trend towards a colder and drier climate as shown by the northern climate indicators from the Bleßberg and Bunker Caves.

### Pearson correlations between demographic and climate data

To statistically evaluate the correlation between the climate and population curves, the Pearson correlation test is applied below.

For this purpose, the demographic proxies (the binned SPDs of the calibrated radiocarbon data) and the palaeoclimate records were synchronized (as best as possible) and separated into 10-year time slices, while the Bunker Cave data were separated into 20-year time slices. Pairwise Pearson correlations between the SPDs of the calibrated radiocarbon data and the palaeoclimate proxies of each region (Circumharz region, Czech Republic/Lower Austria region, Northern Alpine Foreland) were computed at time horizons of 200 (Bleßberg and Spannagel Caves) and 300 years (Bunker Cave), respectively, sliding diachronically from one time slice to the next (e.g., 3550–3360 BCE, 3540–3350 BCE, etc.). Since Bunker Cave and Spannagel Cave have lower δ18O warm/wet climates than Bleßberg Cave, only negative correlations indicate a relationship between warm/wet climate and population growth (Figs 5–7 and S3 Data).

With this statistical correlation, however, it must be kept in mind that a synchronous development between the δ18O curve and the SPD curve cannot always be expected, since a time-delayed anthropogenic response to climate change must be expected. Furthermore, climate determinism should not be assumed, as demographic developments are conditioned by a multitude of different (social, technological, economic, etc.) reasons (cf. Armit et al. [113]).

Figs 5–7 show strong linear (r) Pearson correlations (>+0.5 or <-0.5 and p-value = < 0.025) between climate and SPD data in all regions. These strong correlations are shown as red and blue lines, respectively. The red lines indicate strong Pearson correlations between warm/wet climate and population increase or cold/dry climate and population decrease; blue lines indicate opposite correlations (warm/wet climate and population decrease or cold/dry climate and population increase).

In the Circumharz region, the SPD curve and the population increase between 5500 and 5100 calBP (3550–3150 BCE) correlate strongly with the δ18O curve from Bunker Cave and partially with that from Spannagel Cave. Furthermore, the population decrease around 5000 calBP (3050 BCE) and the increase around 4850 calBP (2900 BCE), which is accompanied by a significant negative deviation in the SPD model, is clearly reflected in the δ18O curves from Bleßberg and Spannagel Caves. The significant strong population increase starting around 4650 BP (2700 calBP) correlates with a warmer and wetter climate. This is shown by strong correlations in all three climate curves (see Fig 5). The population decline in the Circumharz region after 4200 BP (2250 BCE) also shows up in all climate indicators, but they correlate strongly at different times. The data from Spannagel Cave correlate significantly with the SPD curve around 4300 calBP (2350 BCE). Bunker Cave correlates around 4080 calBP (2130 BCE) and the nearby Bleßberg Cave correlates around 3820 calBP (1870 BCE). Opposite developments (warmer/wetter climate = population decline, shown as blue lines in Fig 5) are evident for Spannagel Cave. The fact that from 4150 BP (2200 BCE) an increasingly warm and humid climate is indicated is especially related to the location of Spannagel Cave. According to Fohlmeister, higher δ18O values may reflect an increased precipitation trend during the Late Holocene at high elevation sites [86], pp. 753).

In the Czech Republic/Lower Austria region, the significant population decrease at 5050 calBP (3100 BCE) and the population increase at 4950 calBP (3000 BCE) correlate with the δ18O data from the Bunker and Spannagel Caves. Furthermore, the population increase around 4450 calBP (2500 BCE) resulting in a strong positive deviation is shown by strong correlations and warmer/wetter climates in both Spannagel and Bleßberg Caves. The SPD decrease around 4200 calBP (2250 BCE) is clearly correlated with the climate curve of the Spannagel Cave. The population decline around 3750 calBP (1800 BCE) shows a strong correlation and a colder/drier climate in the Bleßberg Cave. Many opposite trends are shown between the δ18O curve from Bunker Cave and the SPD curve from the Czech Republic/Lower Austria region. The author attributes this to the greater distance and less convergence between these two records.

For the Northern Alpine Foreland, population growth and a warmer/wetter climate are evident in all three climate indicators between about 5450 calBP and 5250 calBP (3500–3300 BCE). The strong Pearson correlations between the nearby Spannagel Cave and the SPD between 5470 and 5270 calBP (3520–3320 BCE) and 4940 to 4530 calBP (2990–2580 BCE) very well illustrate the climatic correlation regarding the establishment and abandonment of the lakeside settlements of the Pfyn and Horgen Cultures and the Goldberg II group. The population increase around 4450 calBP (2500 BCE) correlates with the climate indicator of the Bleßberg Cave, which indicates a significantly warmer and wetter climate. There is also a clear correlation between the δ18O curve of Spannagel Cave and the SPD curve between 4310 and 4020 calBP (2260–2170 BCE). This distinctly shows that the 4.2 ky event is associated with a significant population decline. An opposite trend (warmer/wetter climate and population decline) is evident from about 3850 calBP (1900 BCE) in Bunker Cave and Spannagel Cave.

The linear (r) Pearson correlations between the climate curves and the SPD curves show in many cases the regional relationship between climate and the level of human activity. This is especially true for the phases between 5000–4900, 4200–4100 and 3900–3550 calBP (3050–2950, 2250–2150 BCE and 1950–1600 BCE), which are characterized by colder/drier climates and associated with population declines. Population declines often show up as significant negative deviations in the SPD.

In contrast, the phases between 4850–4650, 4500–4200 and 4000–3900 calBP (2900–2700, 2550–2250 and 2050–1950 BCE) represent warm and humid phases associated with population increases. Population increases often show up as significant positive deviations in the SPD.

However, it remains important to emphasize that there are also opposite developments and that the level of human activity cannot be attributed to climatic conditions alone.

## Discussion

The results presented in this paper provide a picture of long-term regional demographic trends in the regions studied by summarizing the probability distributions of calibrated radiocarbon dates and highlighting the potential impacts of climate change on human populations. In the following discussion, we summarize the causal links between population proxies and the palaeoclimate record, and describe how socio-ecological trajectories varied across the three study regions–the Circumharz region, the Czech Republic/Lower Austria region, and the Northern Alpine Foreland–and across the three archaeological time periods, including–the Late Neolithic, the Final Neolithic, and the Early Bronze Age.

### Late Neolithic

During the Late Neolithic (up to about 4900 calBP, 2950 BCE), different human responses to climatic trends are observed in the study regions.

Around 5500 calBP (3550 BCE), a population boom is observed in all three regions. This is related to the expansion of the cultural groups Salzmünde/Baalberge (Circumharz region), Baden/Boleráz (Czech Republic/Lower Austria region) and Pfyn/Altheim (Northern Alpine Foreland). Especially the Boleráz group of the Baden Culture shows a high and long-lasting population density, which is also shown by the northern distribution of Boleráz elements in Baalberge contexts [43], p. 230). Oxygen isotope curves from the Spannagel Cave and the Bunker Cave indicate a warm winter climate for this phase at the end of the mid-Holocene thermal maximum.

Around 5300 calBP, associated with the Bernburg and Walternienburg groups, the population in the Circumharz region increases. The same is true for the Northern Alpine Foreland and the pile-dwelling settlements of the Horgen group. In the Czech Republic/Lower Austria region, a slight increase in population begins a little later around 5250 calBP. The Bunker Cave indicates a very mild winter climate at this time. The discovery of pottery sherds of the Baden Culture in lakeside settlements in Switzerland (Arbon-Bleiche 3, [43, 114]) indicates a diffusion, probably a migration movement into the Northern Alpine Foreland.

From about 5000 calBP onward, a significant decline of the population in the Circumharz region can be observed. The populations of the Late Neolithic cultural groups of Salzmünde, Walternienburg, and Bernburg decrease. Palaeoclimate records (especially data from the Bleßberg and Spannagel Caves) show strong correlations with a colder and drier climate at this time. An analogous development can also be observed in the Northern Alpine Foreland, which led to a short-lived collapse of the pile dwellings of the Horgen Formation. The Czech Republic/Lower Austria region also shows a significantly lower population density around 5000 calBP (3050 BCE), which, however, shows a renewed increase in population shortly after 5000 calBP (3050 BCE) with the beginning of the Jevišovice and Řivnáč Cultures.

A parallel trend can also be traced outside the study regions for Northern Germany [115, 116]. Here, too, a collapse of human activity around 5000 calBP is detectable, so we assume that this development was a widespread phenomenon.

Furthermore, the Late Neolithic shows a strong convergence with respect to the population development in the Circumharz region and the Northern Alpine Foreland, which may be related to a strong cultural connection or exchange between the two regions. The Czech Republic/Lower Austria region differs in this respect. Based on the similar population dynamics between the Northern Alpine Foreland and the Circumharz region, the Late Neolithic population dynamics for the Northern Alpine Foreland seem plausible, although ^14^C-dates from settlements are strongly overrepresented in this region and time phase compared to ^14^C-dates from grave contexts.

### Final Neolithic

After 4900 calBP (2950 BCE), population density increases in all regions with the onset of the Globular Amphora Culture (Szmyt 2003; Woidich 2014) and the Corded Ware phenomenon. This increase is strongly correlated with the onset of a warm climate. However, between 4600 and 4500 calBP (2650–2550 BCE) a cold episode causes a stagnation (Circumharz region) or a decrease in population, which leads to the demolition of lakeside settlements in the Northern Alpine Foreland. Then, with the Late Corded Ware and Bell Beaker phenomena, a population boom begins, leading to significantly higher population densities, especially in the Circumharz region and in the Czech Republic/Lower Austria region. This increase is strongly correlated with a warm and humid climate, which is particularly evident in the data from the Bleßberg Cave. Due to the black earth soils, the Circumharz region has very good agricultural conditions, and the landscape has been increasingly anthropogenically shaped by large-scale agriculture and pastoralism, which has led to a strong deforestation and expansion of cultivated areas (cf. Meller [39, 117].

Overall, populations are in a growth phase, as conditions are favorable and resources are sufficiently available. However, in the cycle model adapted by Gronenborn [118–120], as the population size increases and resources become limited, the limits of growth are reached. The vulnerability of the system increases and resistance to external influences decreases.

Around 4200 calBP/2250 BCE (4.2 ky event), the palaeoclimate records show a cooling of winter temperatures. Spannagel Cave shows the highest values throughout the study period and correlates strongly with the SPDs for this phase. All SPDs show a population decrease for this phase, which is particularly strong in the Circumharz region and the Northern Alpine Foreland during this transition phase to the Early Bronze Age.

**Fig 9:**
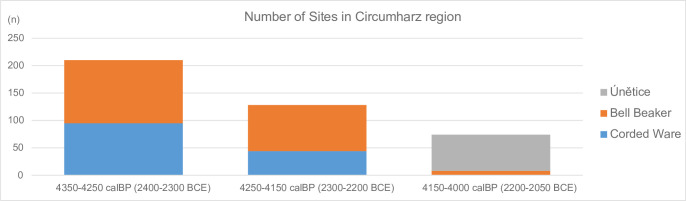
Number of sites and archaeological group assignment in the Circumharz region (focusing on Saxony-Anhalt) quantified according to the maps of Meller (2019, 41, Figs 2.1–2.3).

The decline of the Circumharz SPD is supported by the quantification of the sites in the distribution maps of Meller [39]. This illustrates, analogous to our SPD calculation, the decline of sites from the Final Neolithic to the Early Bronze Age in the Circumharz region (focus on Saxony-Anhalt, [39], p. 41) (Fig 9).

The strong anthropogenic influence, possibly combined with a dry phase, led to wind and water erosion, poorer crop yields, and, especially in the Circumharz region, where a “saturated” system near the capacity limit prevailed, to a population decline [121, 122]. Outside the study region, for example in Northern Germany, a decline in human activity is also evident during this phase [35, 115, 116].

Moreover, the Final Neolithic (as well as the Early Bronze Age) shows strong parallels in population development between the Czech Republic/Lower Austria region and the Northern Alpine Foreland, with a stronger Bell Beaker influence in the Czech Republic/Lower Austria region.

### Early Bronze Age

The Bleßberg and Spannagel Caves indicate a warmer climate from about 4000 calBP (2050 BCE) on, causing an increase in population in the Northern Alpine Foreland and in the Czech Republic/Lower Austria region and a peak of the Southern German Early Bronze Age groups. The situation in the Circumharz region is different. There is no upward trend in the SPD curve. Exactly in the phase of a population increase in the Northern Alpine Foreland and a continued high population level in the Czech Republic/Lower Austria region, a continuous population decrease is observed in the Circumharz region. A phase in which, from about 3900 calBP (1950 BCE), so-called “princely graves” were built in the Circumharz region (Fig 4). The (presumably) last and largest one was built at Bornhöck around 3800 calBP (1850 BCE) (cf. Meller and Schunke [117]).

The question arises whether there is a connection between the bust phase in the Circumharz region and the construction of the so-called “princely tombs”, while in the other two regions there is a boom and recovery phase of the population between 4000 and 3850 calBP (2050–1900 BCE). To explain this asynchronous development, we propose three hypotheses, which are not mutually exclusive and can be intertwined:

(a) We are dealing with a biased data situation. The so-called “princely graves” are associated with an accumulation of settlements. It is possible that in the Circumharz region we are dealing with a process of population accumulation in larger settlements, e.g. Pömmelte/Zwenkau [48, 123–125], which cannot be reflected in the collected ^14^C-dates in such a way, since comparatively few ^14^C-dates from these settlements are available.

It should also be considered that the lack of radiocarbon dates could be related to a more mobile lifestyle and pastoralism. Pastoralists are much harder to trace in the archaeological record than farmers. Increased human mobility and pastoralism, as documented for the Sahel in modern times [126], has been postulated as a consequence of climatic deterioration.

b) The emergence of “princely burials” and the increase of social inequality in the Circumharz region correlate with an unsustainable ecological development and more unfavorable climatic conditions. The strong anthropogenic influence and intensive land use led to changes in the ecosystem and a decrease in agropastoral productivity. At the same time, based on the Bunker and Bleßberg Caves, a cooler/drier climate can be observed from 3900 calBP (1950 BCE) onwards, which led to changes in the ecosystem, to a social crisis as well as to a concentration of power in the hands of a few and to increased social inequality. (cf. Tisdell and Svizzero [121, 122]).

In this context, we would also like to quote Georg Kossack, who points out that challenges such as changing production methods, contact with “higher civilizations”, but also harsh natural conditions, could have encouraged the construction of princely burials [127].

c) The “elites” of the Circumharz region benefited from population increases in neighboring regions. These growths led to increased trade relations and interconnections between the regions presented here. The emergence of fortified settlements in the Czech Republic/Lower Austria region (e.g., Hosty, [128, 129]) and in the Northern Alpine Foreland (e.g., Siedlung Forschner, [130], which could be associated with an increase in bronze technology, exemplifies this development (cf. Langová [131], p. 771; [78, 128]). Only a few actors from the Circumharz region benefited from these trade relations, e.g. those who supplied subsistence goods and the salt originating from the Circumharz region [132] to maintain bronze production.

From 3850 calBP (1900 BCE) onward, a successive population decline can be observed in all study regions. The palaeoclimate record of the Bleßberg Cave shows low δ18O data (rather cold/dry climate) and indicates a moderate to strong influence of the Siberian High and a pronounced negative NAO during this time [87], 158). The result is winter conditions with frequent cold winters and reduced precipitation [112]. During this phase, the δ18O data correlate strongly with the SPDs of the Circumharz region and with the Czech Republic/Lower Austria region. Furthermore, Bunker Cave shows the highest δ18O values of the whole data set around 3800 calBP (1850 BCE), indicating a very cold and dry winter climate. Meanwhile, Spannagel Cave shows an increasingly warmer winter climate, which is related to the altitude of this cave [86], p. 753).

This phase of increased aridity is supported by other archaeological evidence. For example, around 3850 calBP (1900 BCE), more wells of the Únětice group were documented in the Circumharz region [133], pp. 143). Based on the settlement site of Zwenkau, T. Schunke was also able to show that the Únětice wells were dug significantly deeper than the older Corded Ware wells, which in turn would support a change to a more arid regional climate [134], pp. 309–310). It is possible that this climate deterioration, combined with erosion, led to increased crop failure and a steady decline in population.

In the Czech Republic/Lower Austria region, the population decline is less pronounced than in the Circumharz region. This is accompanied by the appearance of fortified settlements of the advanced Early Bronze Age (Bz A2/B1; cf. Langová [131] p. 771). Furthermore, numerous grave finds with amber and genetic analyses suggest that trade routes led to a migration of people from the Baltic Sea [78, 109].

First from about 3550 calBP (1600 BCE), at the beginning of the Middle Bronze Age (cf. Stockhammer et al. [79]), the population size stabilizes, which is particularly evident in the Northern Alpine Foreland. The reason for this could be the development of resilience in the face of these ecological crises. In response to the colder climate, societies introduced spelt, which is considered to be resilient. Evidence exists for Slovakia and Southern Germany. The first evidence of millet, which also grows on drier and more nutrient-poor soils, is also documented, for example, in Slovakia. Diversity and mixing of cultivated and wild plants have also been documented [135–137]. Other resilience strategies include the construction of larger and three-aisled houses, suggesting increased stabling, and the creation of extensive road and trade networks [19, 138], pp. 932–933).

According to the adapted cycle model of Gronenborn [118–120], the Middle Bronze Age is accompanied by a reorganization and renewal of societies. More resources are available again and the vulnerability to external influences in connection with the adaptation of new technologies is lower than before. A new expansion is possible.

## Conclusion

This work has revealed, for the first time, distinct long-term demographic trends in three regions of Central Europe from the Late Neolithic to the beginning of the Middle Bronze Age (5500–3500 calBP/3550–1550 BCE). We used SPDs from calibrated radiocarbon dates to determine population trends in these regions. We also included regional palaeoclimate records to examine the extent to which climate change influenced population trends.

Demographic “booms” in one region were accompanied by “busts” in neighboring regions, raising the possibility that regions prospered–at least in part–at the expense of their neighbors, or that populations migrated from one region to another. This is a basic pattern proposed by Shennan et al. [23] for the European Neolithic.

Our data show parallel population dynamics in the Late Neolithic between the Circumharz region and the Northern Alpine Forelands. During the Final Neolithic and the Early Bronze Age, however, there is a parallel development between the Northern Alpine Foreland and the Czech Republic/Lower Austria region. The Circumharz region differs strongly from the other two regions with regard to the SPD curve of the Early Bronze Age, which might be the result of a biased ^14^C-database.

The results also show statistical correlations between population fluctuations and hydroclimatic patterns. In other words, there was a causal relationship between population trends and climate. This is especially true for the phases between 4850–4650, 4500–4200 and 4000–3900 calBP (2900–2700, 2550–2250 and 2050–1950 BCE). These are warm and wet phases, which increase subsistence economic yields, reduce the risk of crop failure and, consequently, are associated with an increase in population. These phases are characterized by the appearance of the Globular Amphora phenomenon, the boom of the Beaker phenomena (Corded Ware and Bell Beaker) and the establishment and consolidation of Early Bronze Age social systems.

The phases between 5000–4900, 4600–4500, 4200–4100 and 3900–3550 calBP (3050–2950, 2250–2150 and 1950–1600 BCE) are characterized by colder/drier winter climates and are associated with population decline. These phases increase the risk of crop failures, mark upheavals such as the dissolution of Late Neolithic societies, the transition from the Beaker Phenomenon to Early Bronze Age societies and the emergence of so-called “princely burials”, as well as a stratified society with high levels of social inequality.

The authors propose 3 theses to explain the development from 3900 calBP (1950 BCE). The social elite of the Únětice society probably benefited from the upswing in the neighboring regions, which, based on metallurgy, led to increased exchange relations, but also to an increased ecological burden and a decline (or accumulation) in the population of the Circumharz region. From 3900 calBP (1950 BCE) onward, a worsening of the climate (evidenced by the Bunker and Bleßberg Caves) favored the development of a stratified society and the appearance of outstanding burials. From about 3850 calBP (1900 BCE), however, the already stressed ecosystem came to a standstill, leading to a decline in population in all studied regions.

During the Advanced Early Bronze Age, prehistoric communities responded to climatic adversity with technological innovations and adaptation strategies. Strategies include increased housing, the introduction of spelt and millet as more robust grains, and extensive road and trade networks [19, 135–138], pp. 932–933).

In interpreting the summed probability distributions (SPD), however, we also discussed whether there is a bias in the data and whether increases or decreases in the SPD curves are due to different preservation conditions or research traditions.

Ultimately, however, it is important to emphasize that in our attempt to correlate population development and climate change, the level of human activity cannot be solely attributed to climate conditions.

## Supporting information

S1 Data ^14^C dataset related to this paper spanning from 5500 to 2800 uncal yr BP including feature type category and sample material information. (XLSX)

S2 Data Script to reproduce the analyses related to the paper. (R)

S3 Data Pearson correlation of climate and SPD data. (XLSX)
